# GABA Prevents Sarcopenia by Regulation of Muscle Protein Degradation and Inflammaging in 23‐ to 25‐Month‐Old Female Mice

**DOI:** 10.1002/jcsm.13646

**Published:** 2024-11-08

**Authors:** Gunju Song, Hyun‐Ji Oh, Heegu Jin, Hyein Han, Boo‐Yong Lee

**Affiliations:** ^1^ Department of Food Science and Biotechnology, College of Life Science CHA University Seongnam South Korea

**Keywords:** Akt/mTOR/FoxO3a signalling pathway, GABA, inflammaging, muscle, sarcopenia

## Abstract

**Background:**

Sarcopenia is the gradual decrease in skeletal muscle mass, strength and function in elderly individuals. Gamma‐aminobutyric acid (GABA) is a neurotransmitter naturally produced from glutamate by the enzyme glutamic acid decarboxylase. Age‐related decline in GABA is linked to age‐related motor and sensory decline and seems to affect sarcopenia, yet no detailed study has been conducted. In this study, we aimed to investigate the effect of GABA on improving sarcopenia by suppressing muscle protein degradation through supplementing decreased GABA in old mice.

**Methods:**

GABA (10 or 30 mg/kg/day) was orally administered daily to young (3 months) and old (21–23 months) C57BL/6 mice for 7 weeks. The body weight and grip strength of the mice were measured weekly at the same time. After sacrificing the mice, the quadriceps and gastrocnemius muscles were excised from their hind limbs, and the spleen and serum were collected. Histological, biochemical and molecular analyses were conducted in various experiments.

**Results:**

The administration of GABA increased muscle strength (+41%, +70% compared to the aged mouse control group, GABA at doses of 10 or 30 mg/kg/day respectively, *p* < 0.05) and muscle mass (quadriceps: +28%, +46%; gastrocnemius: +12%, +19%, *p* < 0.05) in old mice. This increase was accompanied by a cross‐sectional area (CSA) increase in the quadriceps and gastrocnemius muscle (*p* < 0.05). The administration of GABA increased IGF‐1 levels in serum (*p* < 0.05), leading to the activation of muscle protein synthesis. We found that GABA inhibits sarcopenia by regulating muscle protein degradation through the activation of Akt/mTOR/FoxO3a signalling pathways. GABA also regulates inflammaging, which is a hallmark of age‐related muscle atrophy. There was a significant increase in the F4/80 + CD11b + total macrophage ratio in gastrocnemius and spleen, especially the M1 macrophage ratio increased in old mice. However, GABA administration was effective in suppressing M1 macrophages (gastrocnemius: −40%, − 53%; spleen: −22%, −26%, *p* < 0.05). Pro‐inflammatory cytokines such as TNF‐α and IL‐6, primarily secreted by M1 macrophages, are also decreased by treatment with GABA (TNF‐α: −24%, −27%; IL‐6: −45%, −59%, *p* < 0.05).

**Conclusions:**

Together, this study demonstrates the importance of GABA in maintaining muscle and low‐chronic inflammation during ageing. We suggest that GABA shows potential as a substance that can effectively address sarcopenia and enhance the overall lifespan and well‐being of older individuals.

## 1 Introduction

Skeletal muscle is the largest tissue in the body, accounting for approximately 40%–50% of total body weight, and is responsible for maintaining correct posture and balance [1]. Sarcopenia is a geriatric condition characterised by a progressive loss of skeletal muscle mass, strength and function, which is associated with various health outcomes [2]. This not only decreases mobility and exercise capacity but also increases the risks of complications, falls and death, which are ultimately considered major factors in reducing the quality of life and health of older people. As the ageing population increases worldwide, the number of patients with sarcopenia is also increasing [3]. Therefore, improving sarcopenia should be considered a priority in maintaining a healthy quality of life for the elderly.

Sarcopenia is characterised by an imbalance in muscle protein turnover, with a shift towards increased protein breakdown and reduced protein synthesis [4, 5, 6]. Therefore, inhibiting muscle protein breakdown is considered a fundamental approach to preventing sarcopenia. Thus, inhibiting Foxo3a activity is an effective approach to prevent muscle wasting, as it downregulates MuRF1 (muscle ring finger 1) and Fbx32 (F‐box protein, also known as atrogin‐1), which induce muscle degradation [7, 8]. One of the central pathways for controlling muscle function is the PI3K/Akt pathway, which is modulated by insulin‐like growth factor (IGF‐1) [9]. IGF‐1 signalling has dual roles [10]. It can inhibit muscle atrophy by inhibiting Foxo3a transcription factors and stimulate anabolic processes such as protein synthesis and skeletal muscle hypertrophy through Akt and mTORC1. Stimulation of protein synthesis involves IGF‐1 hormones and activates the PI3K/Akt (phosphoinositide‐3‐kinase/protein kinase B) signalling pathway [11, 12]. This activation leads to the activation of mTOR (mammalian target of rapamycin), which in turn phosphorylates the targets p70S6K (ribosomal p70 S6 kinase) and 4E‐BP1(4E‐binding protein 1) [13]. Therefore, the activation of the IGF‐1/Akt/mTOR pathway in muscles plays an important role in ameliorating sarcopenia by promoting muscle protein synthesis.

In the elderly, chronic and low‐grade systemic inflammation, known as ‘inflammaging’, is linked to many age‐associated diseases [10, 14, 15]. There is a complex interaction between immune cells, especially macrophages, and muscle cells that plays a role in the regeneration and repair of muscle tissue [16]. Dysregulation of this crosstalk can contribute to the development and progression of sarcopenia, observing imbalance in the ratio of pro‐inflammatory (M1) to anti‐inflammatory (M2) macrophages in skeletal muscle [17, 18]. The increase in the proportion of M1 macrophages due to ageing can alter the M1 and M2 ratios, actively contributing to the inflammatory response. This increase in the inflammatory response leads to excessive secretion of inflammatory cytokines, which directly induces muscle protein breakdown [19, 20]. Thus, managing inflammaging through regulating the macrophage ratio is a key strategy for treating sarcopenia in the elderly.

Gamma‐aminobutyric acid (GABA) is a nonproteinogenic amino acid and a naturally occurring neurotransmitter that is synthesised from glutamate through the enzymatic action of glutamic acid decarboxylase [21]. Several animal studies have linked age‐related reductions of GABA, the brain's primary inhibitory neurotransmitter, to age‐related cognitive, motor and sensory decline [22, 23]. While recent research has highlighted the potential benefits of GABA, its specific effects on sarcopenia remain inadequately explored. Noteworthy are the various biological activities attributed to GABA, such as its antioxidant, stress‐alleviating and sleep‐promoting properties [24, 25, 26]. This study aimed to evaluate whether GABA could impede the onset and progression of sarcopenia in 23‐ to 25‐month‐old mice by oral administration to replenish the decreased production of GABA.

## 2 Methods

### 2.1 Reagents

GABA (A5835; Sigma‐Aldrich, St. Louis, MO, USA) obtained from Amorepacific Corp. was dissolved in distilled water and administered to each mouse via oral gavage at doses of 10 or 30 mg/kg/day. The dose of GABA in this study was determined after consulting several studies that reported multiple bioactive effects [27, 28, 29, 30].

For flow cytometry analysis, antibodies specific for FITC anti‐mouse CD11b, AF647 anti‐mouse F4/80, PE anti‐mouse CD163 and PerCP/Cyanine5.5 anti‐mouse CD206 were purchased from BioLeged (San Diego, CA, USA). Antibodies specific for phospho‐forkhead box O3 (p‐FoxO3a), FoxO3a, phospho‐Akt (p‐Akt), Akt, phospho‐mammalian target of rapamycin (p‐mTOR), mTOR, phospho‐4EBP1, 4EBP1, phos‐pho‐p70S6 kinase (p‐p70S6K) and p70S6K, inducible nitric oxide synthase (iNOS) were purchased from Cell Signalling Technology (Danvers, MA, USA). Antibodies specific to the F‐box protein (Fbx32/atrogin), muscle ring finger 1 (MuRF1) and myoblast determination protein 1 (MyoD) were purchased from Abcam (Cambridge, UK). Antibodies specific to phosphoinositide 3‐kinase (PI3K), myogenin, myocyte enhancer factor 2 (MEF‐2), cy‐clooxygenase‐2 (COX‐2) and glyceraldehyde 3‐phosphate dehydrogenase (GAPDH) were purchased from Santa Cruz Biotechnology (Dallas, TX, USA).

### 2.2 Animals and Treatments

The 3‐month‐old or 21‐ to 23‐month‐old female C57BL/6J mice were purchased from the Korea Research Institute of Bioscience and Biotechnology (Daejeon, Korea). The establishment of aged mouse models involves breeding mice until they reach 20 months of age. Female mice were selected over males because they are less aggressive, making them easier to handle and age. All animals were cared for according to the standards outlined in the ‘Guide to the Care and Use of Laboratory Animals’ prepared by the National Academy of Science and published by the National Institutes of Health. Animal experiments were approved by the Institutional Animal Care and Use Committee (IACUC) of CHA University (IACUC210145). Mice were housed at 20 ± 3°C in a room maintained under a 12‐h light–dark cycle 1 week prior to administration for adaptation. Mice were randomly assigned to five groups (*n* = 10/group) as follows: (i) young mice aged 3‐month‐old control group (YM Ctrl), (ii) young mice administered 30 mg/kg/day of GABA (YM GABA30), (iii) old mice aged 21‐ to 23‐month‐old control group (OM Ctrl), (iv) old mice administered 10 mg/kg/day of GABA (OM GABA10) and (v) old mice administered 30 mg/kg/day of GABA (OM GABA30). Each concentration of GABA was administered to the mice for 7 weeks. At the end of the treatment period, the mice were sacrificed, and all samples were weighed and stored at −80°C for further analysis.

### 2.3 Measurement of Body Weight and Grip Strength

The mice body weight and grip strength were measured 1 day before the start of administration and once a week for 7 weeks during the administration period. A Chatillon force measurement system (Columbus Instruments, Columbus, OH, USA) was used to measure all‐limb grip strength. The mouse was placed on the mesh grid, allowing the mouse to grasp the mesh with their four feet, and then the tail was gently pulled three to five times to measure the force just before the mouse fell off the grid. The average grip force of the mouse limbs was recorded.

### 2.4 Histological Analysis

The quadriceps and gastrocnemius muscles were accurately isolated from the hind legs of mice, fixed in 4% paraformaldehyde, embedded in paraffin and sectioned into 10‐μm slices. Sections were stained with haematoxylin and eosin (H&E) for histological analysis. The stained muscle tissues were imaged under a Nikon E600 microscope (Nikon, Tokyo, Japan). The cross‐sectional area (CSA) of muscle fibres was measured using ImageJ software (Bethesda, MD, USA).

### 2.5 Western Blotting

The quadriceps and gastrocnemius muscle samples were minced and lysed for 30 min in lysis buffer (iNtRON Biotechnology, Seoul, Korea) containing protease and phosphatase inhibitors. Proteins in the lysate were quantified using a BCA protein assay (Pierce, Rockford, IL, USA). Equal amounts of proteins were electrophoresed using SDS‐PAGE and transferred to Immun‐Blot PVDF membranes (Bio‐Rad, Hercules, CA, USA). After 1 h of blocking in 5% skimmed milk, the membranes were washed with Tris buffered saline containing 0.05% Tween‐20 and then incubated overnight at 4°C with primary antibodies. The membranes were washed and blocked for 1 h in 5% skimmed milk containing secondary antibody (peroxidase‐conjugated antirabbit, antimouse or antigoat antibodies, Bio‐Rad, Hercules, CA, USA). The protein signal intensities were detected using an EZ‐Western Lumi Femto kit (DoGenBio, Seoul, Korea) and imaged using a LAS‐4000 apparatus (GE Healthcare Life Sciences, Marlborough, MA, USA). The relative band intensities were quantified and calculated using ImageJ software (Bethesda, MD, USA).

### 2.6 Serum Analysis

After 7 weeks of oral administration, blood samples were obtained via cardiac puncture from mice at the time of sacrifice. The samples were centrifuged at 800 × g for 15 min at 4°C, and the serum was stored at −80°C for subsequent analysis. Serum levels of IL‐6 and TNF‐α were evaluated using a Mouse High Sensitivity T Cell Magnetic Bead Panel (Merck Millipore, MA, USA). Serum levels of (IGF)‐1 were measured using an IGF‐1 ELISA kit (Thermo Fisher Scientific, MA, USA). All analyses were performed according to the manufacturers' instructions. Absorbances were measured at appropriate wavelengths using a Luminex 100 analyser (Luminex, Austin, TX, USA). All samples were assessed in triplicate.

### 2.7 Flow Cytometry Analysis

Spleen samples from the mice were minced, and single cells were obtained by passing the splenocytes through a 40‐μm strainer. Red blood cells were dissolved in ammonium–chloride–potassium lysis buffer (Lonza, Basel, Switzerland). After washing, single cells were stained with specific antibodies for 30 min on ice to observe total macrophages (CD11b+F4/80+), M1 (CD11b+F4/80+CD163‐CD206‐), M2a (CD11b+F4/80+CD163‐CD206+) and M2c (CD11b+F4/80+CD163+CD206+) macrophages. Gastrocnemius muscle samples were minced and incubated in collagenase and Dispase II (Sigma‐Aldrich, St. Louis, MO, USA) at 37°C for 1 h. The tissues were dissociated by passing them through a 40‐μm strainer. Single cells were stained with specific antibodies for 30 min on ice to observe total macrophages (CD11b+F4/80+), M1 (CD11b+F4/80+CD163‐CD206‐), M2a (CD11b+F4/80+CD163‐CD206+) and M2c (CD11b+F4/80+CD163+CD206+) macrophages.

Flow cytometry was performed using a CytoFlex flow cytometer (Beckman Coulter, CA, USA), and the data were analysed with FlowJo software (Ashland, OR, USA).

### 2.8 Statistical Analysis

All parameters are expressed as the mean ± standard error of the mean (SEM), and the comparisons were estimated using a one‐way analysis of variance (ANOVA) followed by Scheffe's test. *p* < 0.05 was considered significant, and values with different letters are statistically different (a > b > c > d).

## 3 Results

### 3.1 GABA Improves the Strength, Mass and Size of Skeletal Muscle in Old Mice

**FIGURE 1:**
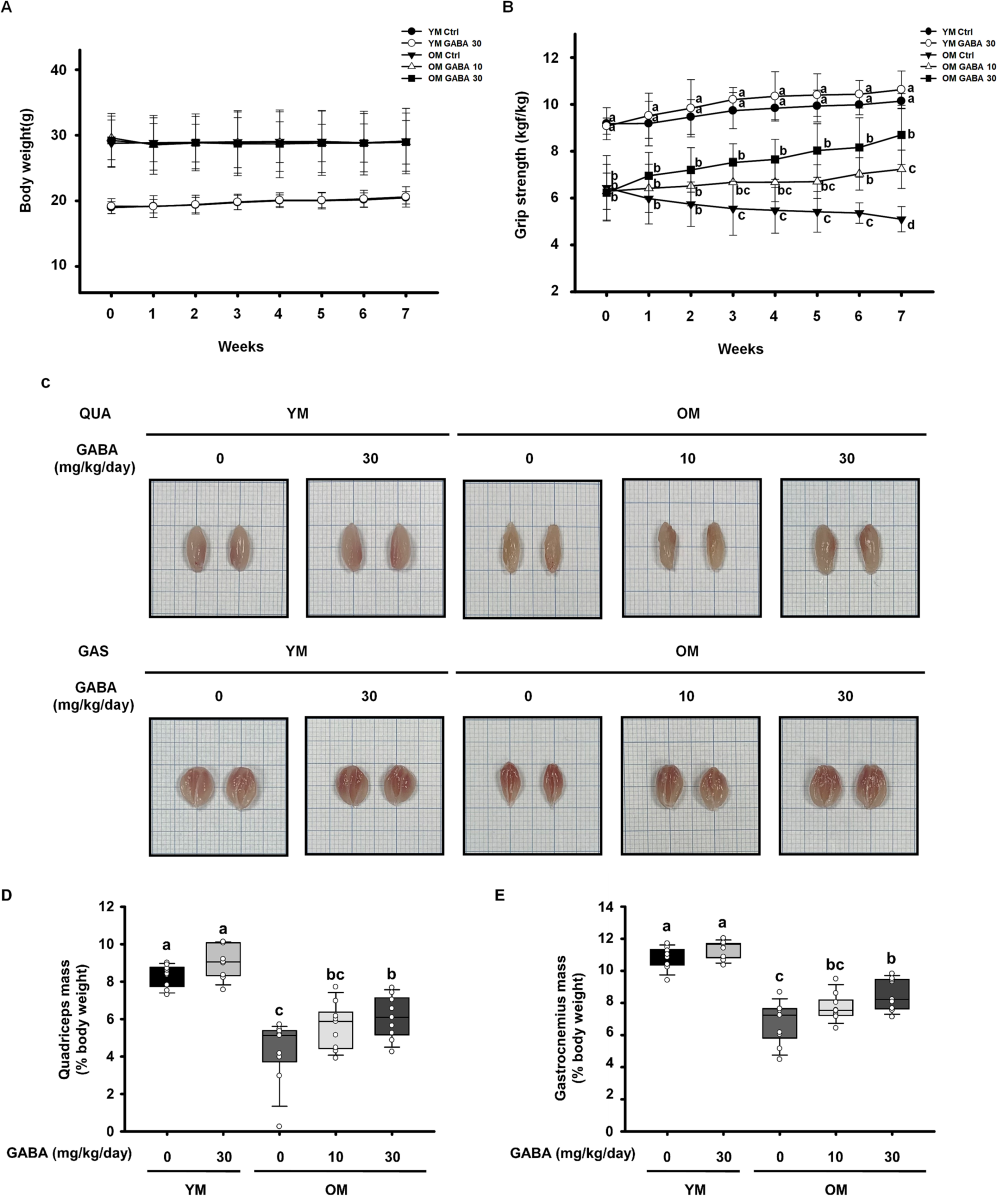
Effect of GABA on muscle strength, mass and size in old mice. (A) The body weight (*n* = 8) and (B) grip strength (*n* = 7) of mice were measured for 7 weeks. (C) Representative images of the QUA (quadriceps, top) and GAS (gastrocnemius, bottom) muscles. The muscle mass of the (D) quadriceps (*n* = 9) and (E) gastrocnemius (*n* = 9). Muscle mass was standardised to the body weight on the last day. Different letters indicate statistically significant differences; *p* < 0.05, a > b > c.

Sarcopenia is characterised by age‐related loss of muscle mass, strength and function [31]. To evaluate the effect of GABA on muscle function and overall health, young (3‐month‐old) and old (21‐ to 23‐month‐old) mice were treated with GABA (10 or 30 mg/kg/day) for 7 weeks. Throughout the study period, the body weight and grip strength of the mice were measured weekly at the same time. Body weight, a fundamental health parameter, remained stable in the control groups as well as in the OM and YM groups that received GABA during the treatment period (Figure 1A). However, there was a notable variation in body weight relative to age observed between the young mice group (YM control, YM GABA30) and the old mice group (OM control, OM GABA10, OM GABA30). Therefore, measurements of grip strength and muscle weight were normalised to body weight. During the experiment period, grip strength was significantly lower in the old mice group than in the young mice group. During the 7‐week treatment period, the OM control group exhibited a gradual decrease, indicating the progression of sarcopenia associated with ageing. However, treatment with GABA improved the grip strength in the OM GABA10 and OM GABA30 groups over the 7 weeks, respectively (Figure 1B). As shown in Figure 1C, the muscles from the OM control group were smaller than those from the YM groups, but treatment with GABA slightly increased muscle size in the OM GABA10 and OM GABA30 groups (Figure 1C). The muscle mass of the OM group was significantly lower than that of the YM group, and muscle mass improved with GABA administration in old mice (Figure 1D,E). Taken together, these data indicate that 7 weeks of GABA administration ameliorates the sarcopenia of old mice by increasing their muscle strength and mass, without affecting their body weight.

### 3.2 GABA Increases Muscle Fibre Size and Myogenic Transcription Factors in old Mice

**FIGURE 2:**
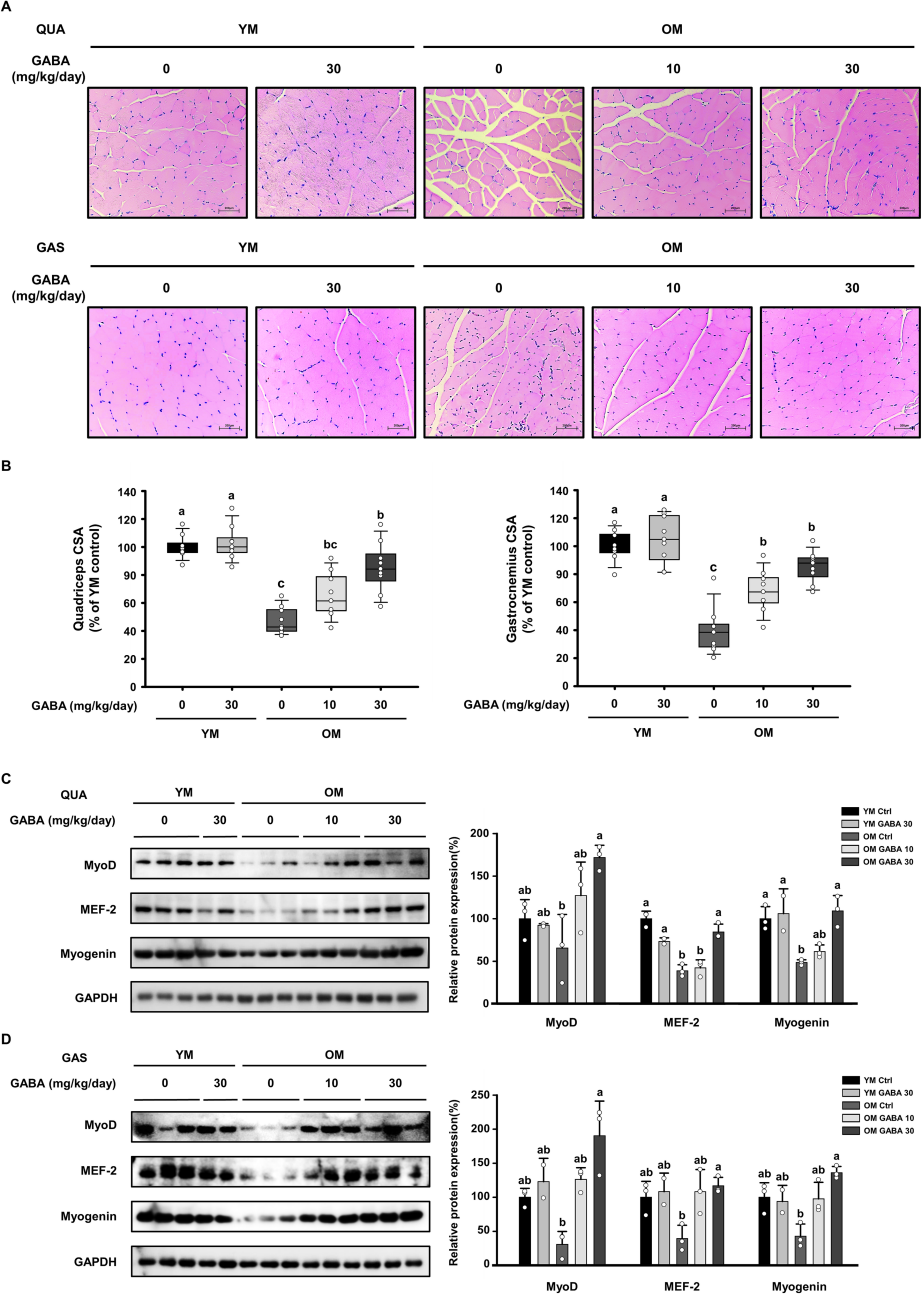
Effect of GABA on muscle fibre size and the expression of myogenic transcription factors in old mice. (A) Histological analysis of haematoxylin and eosin staining of the QUA (top) and GAS (bottom) muscles. (B) Cross‐sectional area (CSA) of the quadriceps (*n* = 9) and gastrocnemius (*n* = 9) muscles. The protein expression of myogenic transcription factors was measured by Western blotting in the (C) QUA and (D) GAS muscles. GAPDH was used as a control. Different letters indicate statistically significant differences; *p* < 0.05, a > b > c.

We analysed the CSA of the quadriceps and gastrocnemius muscles to determine if the above results were associated with increased fibre size. Histological analysis revealed that the mean muscle fibre size was smaller in the OM control group compared to the YM groups, and treatment with GABA prevented the decline in muscle fibre size associated with ageing (Figure 2A). Each muscle fibre CSA showed a significant recovery in the OM GABA10 and OM GABA30 groups (Figure 2B). We also measured the expression of myogenic transcription factors in the quadriceps and gastrocnemius muscles of each group to determine whether GABA promotes myogenesis. As a result, the protein expression of myoblast determination protein 1 (MyoD), myocyte enhancer factor 2 (MEF‐2) and myogenin in both muscles was lower in the OM control group than in the YM group. However, the expression of these proteins in the OM GABA10 and OM GABA30 groups increased with the treatment of GABA (Figure 2C,D). These data suggest that the treatment of GABA induces myogenesis by promoting the expression of myogenic transcription factors, which subsequently improves age‐induced decrease in muscle fibre size in old mice.

### 3.3 GABA Activates the IGF‐1/Akt/mTOR Signalling Pathway in Old Mice

**FIGURE 3:**
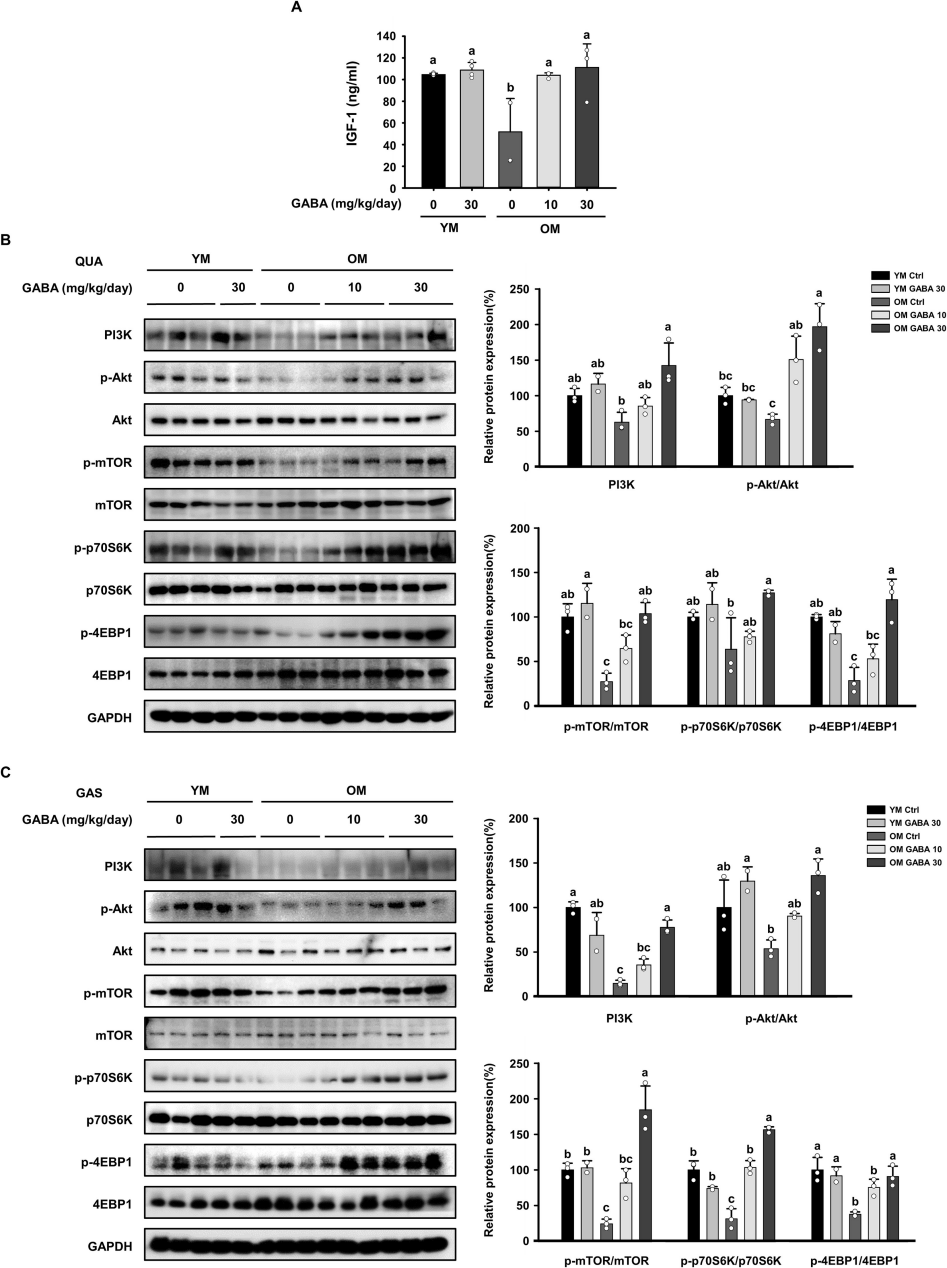
Effect of GABA on the Akt/mTOR signalling pathway in old mice. (A) Serum levels of IGF‐1 (*n* = 4). The protein expression levels of Akt/mTOR signalling pathway‐related factors were examined by Western blotting in the (B) QUA and (C) GAS muscles. GAPDH was used as a control. Different letters indicate statistically significant differences; *p* < 0.05, a > b > c.

Muscle mass is also increased through the activation of the IGF‐1/Akt/mTOR signalling pathway. The serum concentration of IGF‐1, which activates the Akt/mTOR signalling pathway and thereby upregulates muscle protein synthesis, tended to be lower in the OM control group than in the YM control group. However, it was increased by the treatment of GABA in old mice (Figure 3A). Next, we investigated the effect of GABA on the activation of the Akt/mTOR signalling pathway by analysing the quadriceps and gastrocnemius muscle tissues. The protein expression of PI3K, which is activated by IGF‐1, was higher in the YM groups than in the OM control group. However, treatment with 10 and 30 mg/kg/day of GABA significantly increased the protein expression of PI3K and the p‐Akt/Akt ratio in both muscles of the old mice (Figure 3B,C). When PI3K/Akt is activated and mTOR is stimulated, the p70S6K and 4E‐BP1 promote protein synthesis through phosphorylation. In both muscles of the OM control group, the p‐mTOR/mTOR ratio was significantly lower than in the YM groups, but the ratios were increased by the treatment with GABA (Figure 3B,C). The lower levels of activation of mTOR in the OM control group tend to correspond with the low phosphorylation levels of the downstream mediators 4E‐BP1 and p70S6K. However, GABA treatment resulted in an increase in the p‐mTOR/mTOR, p4E‐BP1/4E‐BP1 and p‐p70S6K/p70S6K ratios (Figure 3B,C). These results indicate that GABA treatment restores age‐related deficiencies, such as Akt/mTOR signalling associated with protein synthesis in the skeletal muscle of old mice and IGF‐1 hormone levels, which are upstream.

### 3.4 GABA Decreases the Expression of FoxO3a and E3 Ubiquitin Ligases in Old Mice

**FIGURE 4:**
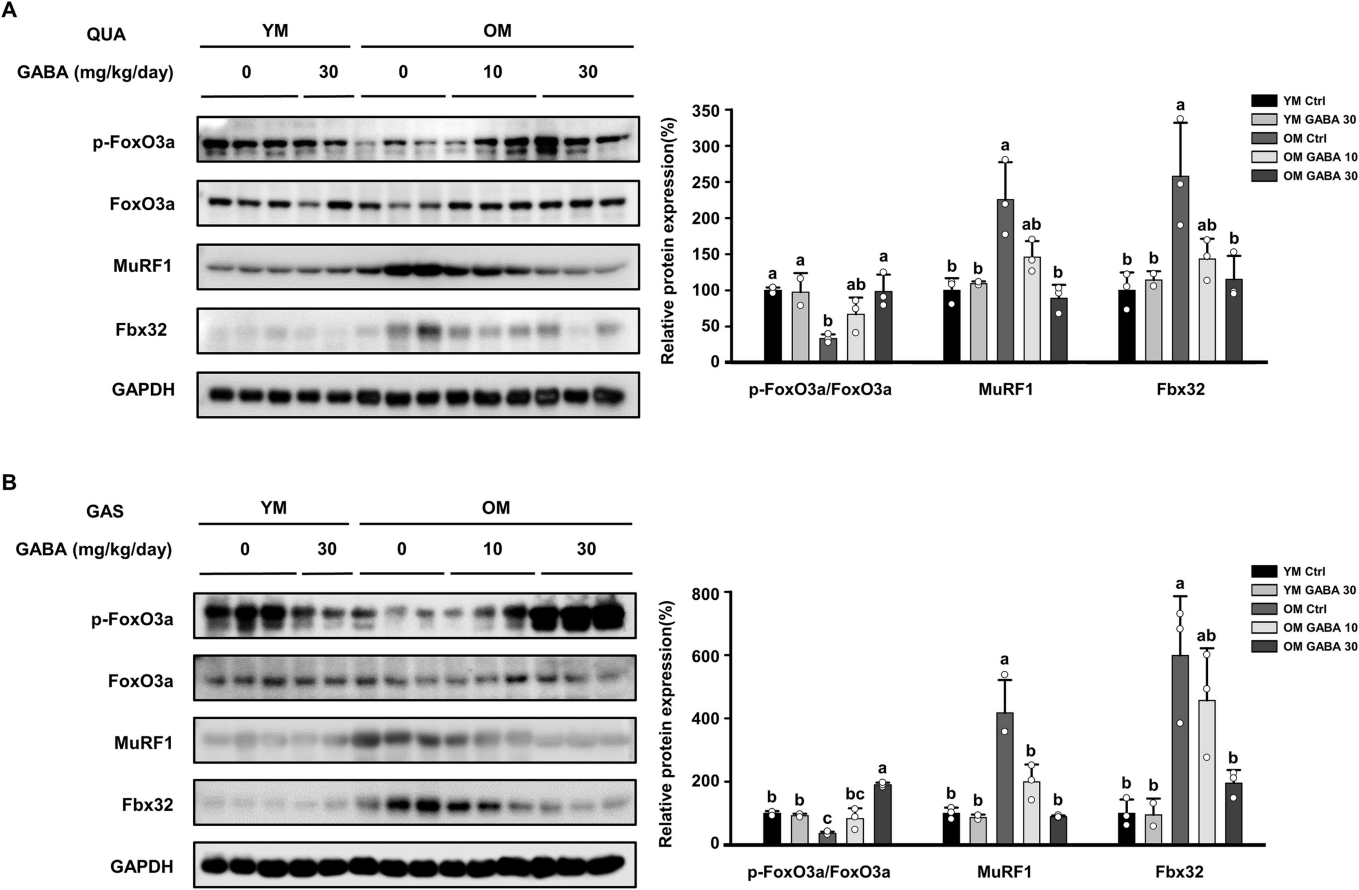
Effect of GABA on the expression of FoxO3a and E3 ubiquitin ligases in old mice. The protein expression levels of p‐FoxO3a, FoxO3a and E3 ubiquitination‐related factors (MuRF1, Fbx32) were assessed by Western blotting in (A) QUA and (B) GAS muscles. GAPDH was used as a control, and the phosphorylation of FoxO3a was normalised to the total protein expression. Different letters indicate statistically significant differences; *p* < 0.05, a > b > c.

FoxO is a transcription factor that plays a role in regulating the transcription of genes associated with the ubiquitin–proteasome system. When phosphorylated, FoxO3a, in its inactive state, is unable to translocate into the nucleus and thus remains localised in the cytosol. Consequently, it is unable to influence the expression of genes that encode components of the ubiquitin–proteasome system. The protein expression of phosphorylated FoxO3a, which was reduced in the OM control group compared to the YM groups, was upregulated in response to GABA treatment in both muscles. This result suggests that GABA treatment prevents FoxO3a from translocating to the nucleus by increasing its phosphorylation. Therefore, the protein expression level of p‐FoxO3a/FoxO3a in the quadriceps and gastrocnemius muscles of the OM Control group was decreased compared to that of the YM group (Figure 4A,B). The expression of Fbx32 and MuRF1 was higher in both muscles of the OM control than in the YM group. However, GABA treatment significantly reduced the expression of both proteins following the expression of p‐FoxO3/FoxO3a (Figure 4A,B), indicating that GABA can effectively reduce skeletal muscle loss by inhibiting protein ubiquitination in old mice. Taken together, these data demonstrate that GABA significantly reduces muscle loss by blocking ubiquitin–proteasome signalling mediated by FoxO3 in old mice.

### 3.5 GABA Suppresses Inflammaging Through the Regulation of Macrophage Subpopulations in Old Mice

**FIGURE 5:**
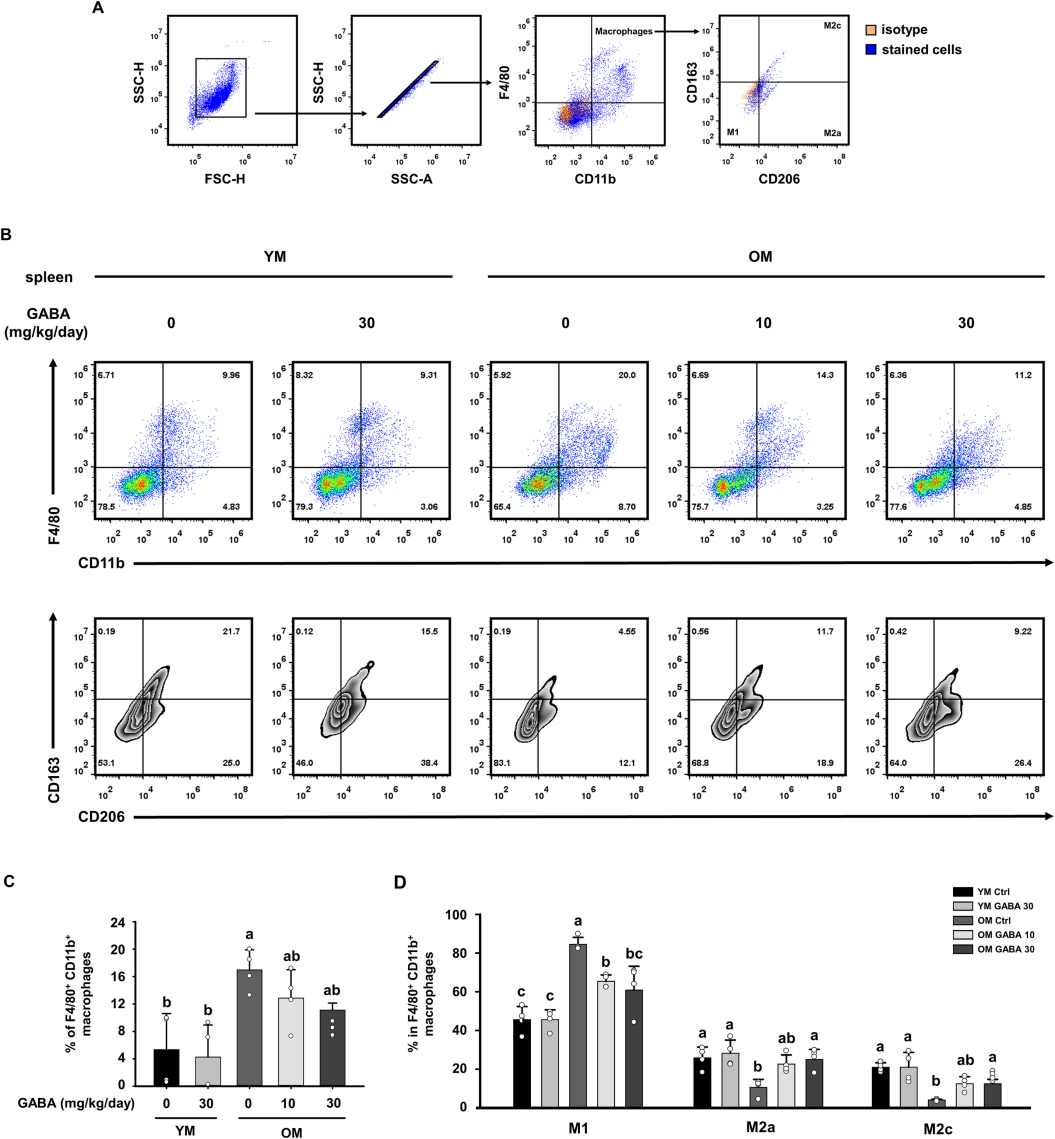
Effect of GABA on the macrophage ratio in the spleen of old mice. (A) Gating strategy for the analysis of macrophage subsets. We gated out debris in the SSC (side scatter) vs FSC (forward scatter) plots; then gated on singlets, live cells and F4/80+ and CD11b+ cells and finally identified subpopulations using CD163+ and CD206+ markers. Orange dots represent the isotype controls, and blue dots represent the cells dyed with specific antibodies. (B) Representative flow cytometry dot plots of CD11b+ F4/80+ macrophages and CD163‐CD206‐M1, CD163‐CD206+M2a and CD163+CD206+M2c macrophages. (C) Bar graph showing the mean percentage of CD11b+ F4/80+ macrophages in the spleen (*n* = 4). (D) Bar graph showing the mean percentage of M1, M2c, and M2a macrophages in CD11b+ F4/80+ macrophages (*n* = 4). Different letters indicate statistically significant differences; *p* < 0.05, a > b > c.

The term ‘inflammaging’ refers to systemic, chronic, low‐grade inflammation observed in elderly individuals, which has been associated with characteristic signs of muscle loss, decreased strength and diminished functionality known as sarcopenia [32]. Macrophages are essential in both the initiation and resolution of inflammation, and their altered function with age, which significantly contributes to the phenomenon known as inflammaging [33]. Therefore, we investigated whether the treatment of GABA has an anti‐inflammaging effect by regulating the macrophage ratio in old mice. First, we investigate the effects of GABA on M1 macrophage phenotype changes in the spleen. Cell phenotypes were defined as follows: M1 macrophages as CD206‐CD163 cells, M2a macrophages as CD206+ CD163‐ cells, and M2c macrophages as CD206+ CD163+ cells, gated on F4/80+ CD11b+ total macrophage cells (Figure 5A). The CD11b+F4/80+ macrophage ratio in the spleen was significantly higher in the OM control group compared to the YM group but decreased in the GABA‐treated OM group (Figure 5B,C). As a result of changes in macrophage subpopulations, M1 macrophages increased in the OM control group compared to the YM group, while M2c and M2a macrophages decreased. The administration of GABA alleviates the relatively high M1 population in the old mice and restores the ratio of M2c and M2a macrophage populations (Figure 5B,C).

**FIGURE 6:**
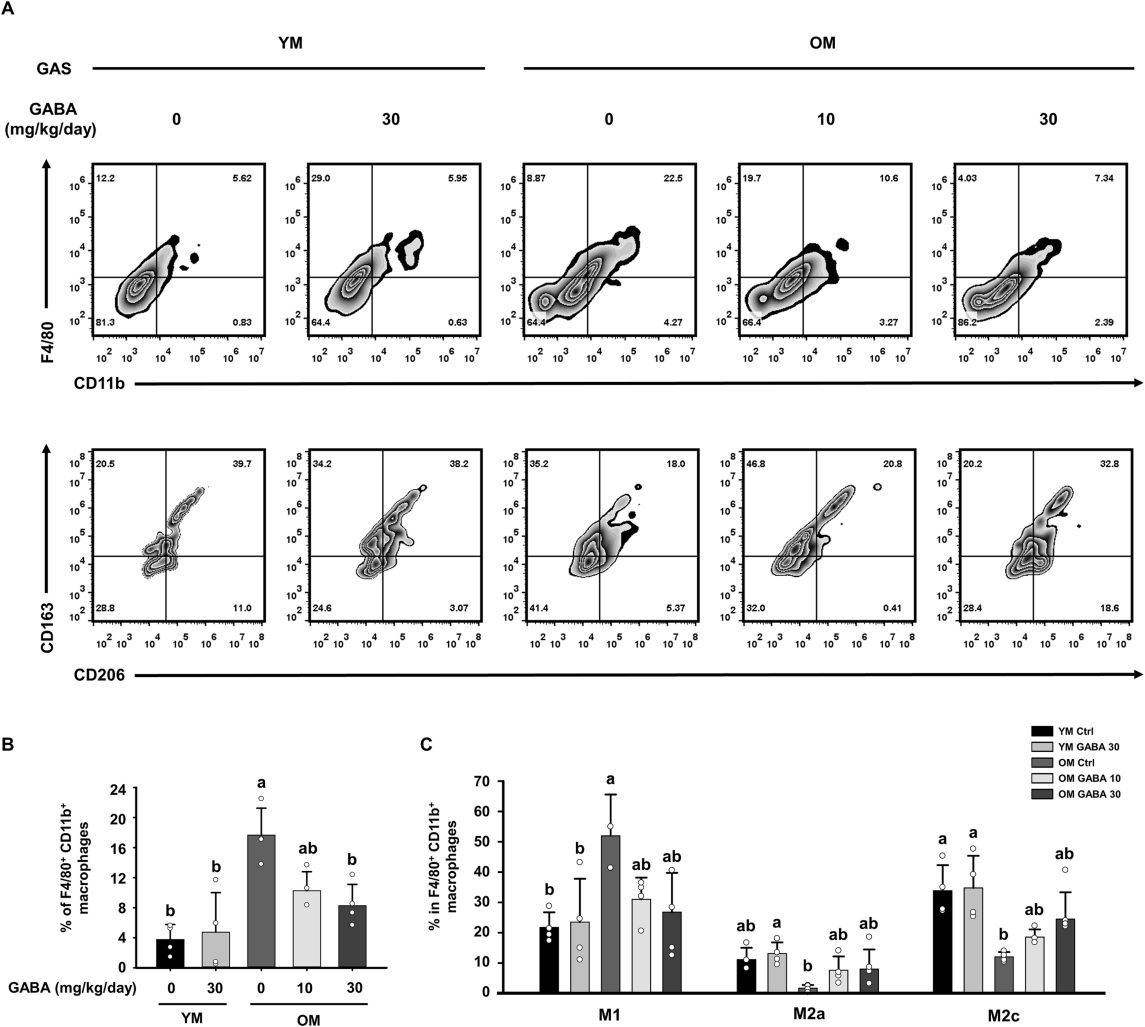
Effect of GABA on the macrophage ratio in the gastrocnemius muscle of old mice. (A) Representative dot plots of CD11b+F4/80+ macrophages and CD163‐CD206‐M1, CD163‐CD206+M2a and CD163+CD206+M2c macrophages. (B) Bar graph showing the mean percentage of CD11b+F4/80+ macrophages in the gastrocnemius muscle (*n* = 4). (C) Bar graph showing mean percentage of M1, M2c and M2a macrophages in CD11b+ F4/80+ macrophages (*n* = 4). Different letters indicate statistically significant differences; *p* < 0.05, a > b > c.

We next investigated whether GABA affected changes in macrophage subpopulations infiltrating the gastrocnemius muscle tissue to confirm the inflammatory state that directly affects muscle degradation. CD11b+F4/80+ macrophages in the gastrocnemius muscle were significantly increased in the OM Control group compared to the YM groups but decreased in the GABA‐treated OM groups (Figure 6A,B). As a result of changes in the ratio of macrophage subpopulations, M1 macrophages increased in the OM control group compared to the YM control group, while M2c and M2a macrophages decreased. The increase in F4/80+ CD11b+ total macrophages in the OM Ctrl group, especially the increase in M1 macrophages, provides substantial evidence of the development of an inflammaging state in old mice muscle. However, the administration of GABA showed a similar proportion of M1 macrophages (CD163‐CD206‐) to the YM groups. In addition, the ratio of M2a macrophages (CD206+CD163‐) and M2c macrophages (CD206+CD163+) significantly increased in response to the GABA treatment (Figure 6A,C). Taken together with the above findings, it suggests that GABA improved inflammaging, which caused muscle loss by directly enhancing the proportion of macrophages in the gastrocnemius muscle.

### 3.6 GABA Attenuates Pro‐Inflammatory Cytokine Levels and Inflammatory Responses in Old Mice

**FIGURE 7:**
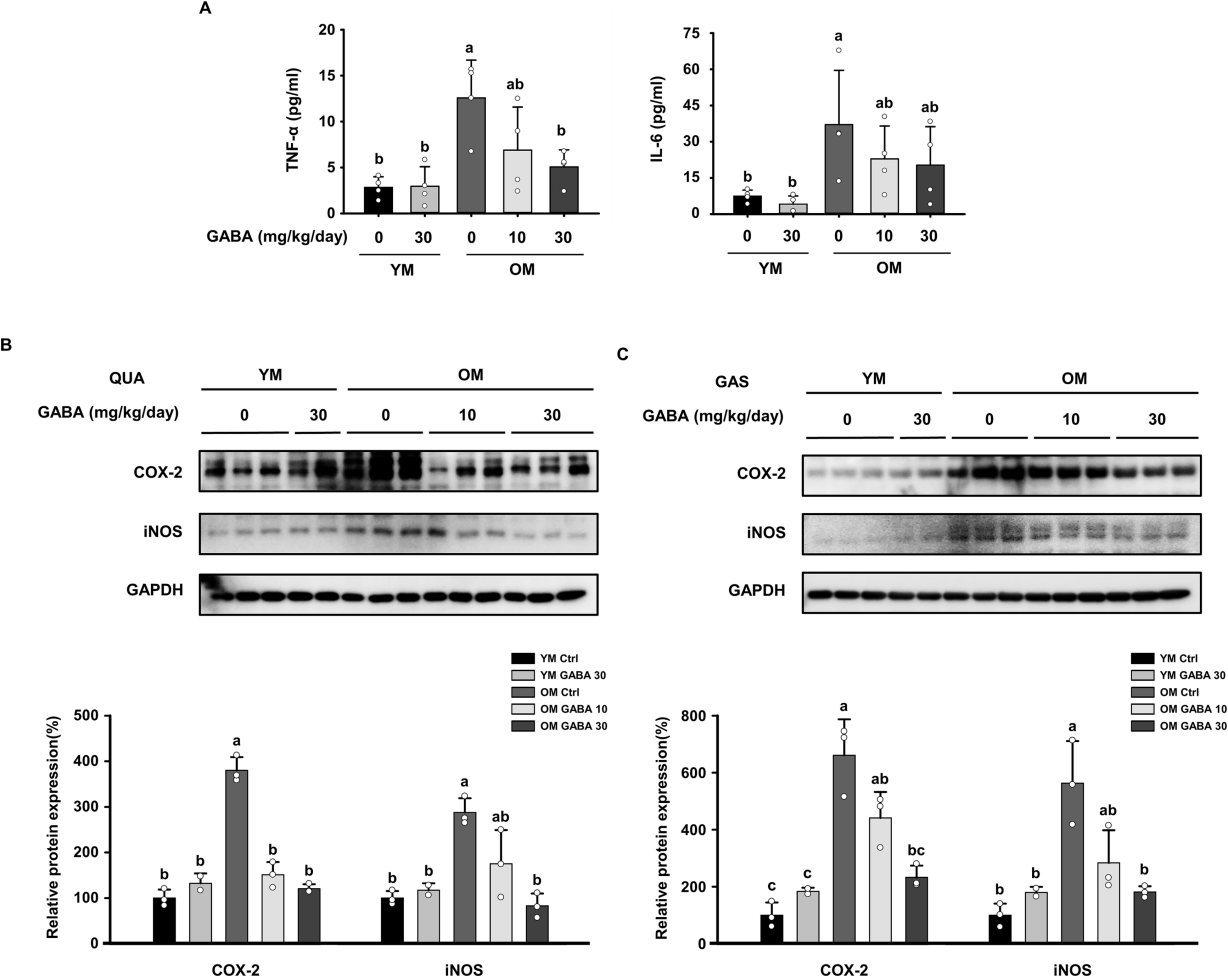
Effect of GABA suppresses age‐related inflammatory responses. (A) Levels of pro‐inflammatory cytokines (TNF‐α, IL‐6) in the serum (*n* = 4). Expression of proteins involved in inflammation in the (B) GAS and (C) QUA muscles was measured by Western blotting. GAPDH served as a control. Different letters indicate statistically significant differences; *p* < 0.05, a > b > c.

In addition to regulating macrophages in the spleen and gastrocnemius muscles, we measured the pro‐inflammatory cytokines TNF‐α and IL‐6, which are representative inflammatory markers, in the serum. The pro‐inflammatory cytokines IL‐6 and TNF‐α, secreted by M1 macrophages, directly cause muscle loss by facilitating the degradation of myofibrillar protein [34]. The serum levels of IL‐6 and TNF‐α were significantly higher in the OM control group compared to the young control group, indicating that old mice were experiencing low‐grade chronic inflammation (Figure 7A). However, GABA treatment significantly reduced the serum levels of pro‐inflammatory cytokines in the OM GABA10 and OM GABA30 groups. As pro‐inflammatory cytokines circulate in the blood and reach the muscle cell membrane, they induce inflammation and increase the expression of COX‐2 and iNOS. In both the quadriceps and gastrocnemius muscles, the expression of COX‐2 and iNOS proteins in the OM control group was significantly higher than that in the young control group, indicating that old mice were experiencing a state of low‐grade chronic inflammation (Figure 7B,C). However, the expression of both inflammatory markers significantly decreased after GABA treatment in old mice. Overall, these data suggest that GABA treatment suppresses age‐induced chronic inflammation and restores muscle homeostasis in old mice.

## 4 Discussion

As life expectancy continues to increase, the age‐related decline in muscle mass and strength, known as sarcopenia, is becoming more evident [31]. Consequently, it has a more significant impact on society. In the present study, the data showed significantly lower parameters related to muscle mass, strength, fibre size and muscle‐specific biomarkers in the 23‐ to 25‐month‐old mice than in the 5‐month‐old mice. As demonstrated in the results of this study, the main characteristic features of sarcopenia include the loss of skeletal muscle mass and a gradual decrease in muscle strength. However, treatment with GABA for 7 weeks in old mice improved muscle strength while maintaining body weight. GABA also increased the size and mass of the quadriceps and gastrocnemius muscles, which are the large muscles of the mouse's hindlimbs that represent overall muscle mass, in old mice. In addition, both hindlimb muscles exhibited an increase in CSA and muscle strength with GABA administration. This finding systematically describes the positive impact of restoring depleted GABA levels in aged mice on sarcopenia.

In this study, we investigate whether GABA has the effect of promoting muscle protein synthesis and, more importantly, whether it significantly inhibits muscle protein breakdown. The results showed that GABA significantly inhibited ubiquitin–proteasome signalling mediated by FoxO3a in old mice. The elderly generally have decreased IGF‐1 levels with age, which may result in reduced activation of downstream pathways, which can lead to muscle loss and decreased muscle function [11]. Thus, proper activation of the IGF‐1/Akt/mTOR pathway plays an important role in preventing sarcopenia and maintaining muscle health [12]. In young mice skeletal muscle, we found that the IGF‐1/Akt/mTOR signalling pathway represses the MuRF1 and Fbx32 genes by reducing the expression and activity of Foxo3a transcription factors. As muscles age, IGF‐1 levels decreased, Foxo3a transcription factors activate the expression of MuRF1 and Fbx32, thereby promoting muscle atrophy. In the present study, GABA treatment restored the levels of IGF‐1 and activities of the Akt/mTOR pathway in both muscles of old mice. These results imply that GABA contributed to stimulating muscle protein synthesis by rescuing the Akt/mTOR signalling pathway in old mice. Taken together with the above results, we suggest that GABA exerts a regulatory effect on muscle protein degradation by regulating the Akt/mTOR/FoxO3a signalling pathway. However, further studies are needed to elucidate the underlying mechanisms of how GABA could increase IGF‐1 in old mice. Furthermore, the expression levels of MyoD, which regulates skeletal muscle differentiation, and MEF‐2 and myogenin, muscle synthesis regulators, were much higher in the young mice groups than in the old mice control group [35, 36, 37]. However, in old mice, the expression of myogenic transcription factors increased due to GABA, resulting in the restoration of muscle fibre size. We demonstrated the effectiveness of GABA in improving sarcopenia by highlighting its role in promoting muscle synthesis and especially in inhibiting muscle degradation.

The term ‘inflammaging’ refers to systemic, chronic, low‐grade inflammation observed in many elderly individuals, which is a hallmark of sarcopenia and a potential target for therapeutic approaches. A growing number of studies are highlighting ‘inflammaging’ as a crucial regulator of the mechanisms that control skeletal muscle homeostasis, ultimately leading to sarcopenia [10, 38]. Indeed, age‐related factors such as cellular senescence, oxidative stress, mitochondrial dysfunction, reduced numbers and hormonal and cytokine changes all play a significant role in the development of sarcopenia. For its part, and importantly, the immune system plays a pivotal role in controlling all these age‐related features [16]. Thus, muscle wasting is a multifactorial condition in which a feedback process occurs between different factors, with inflammation at its core. As one of the representative progresses of inflammaging, an imbalance between M1 and M2 macrophage patterns was observed in ageing [39, 40]. Indeed, we confirmed an increase in M1 macrophages accompanied by a decrease in M2 macrophages in old mice through flow cytometry analysis. However, GABA administration was effective in suppressing inflammaging by regulating the ratio of M1 and M2 macrophages. This result shows that GABA inhibits inflammaging in the spleen and in the gastrocnemius muscles, which are the most direct tissues affected by sarcopenia. Pro‐inflammatory cytokines such as TNF‐α and IL‐6, primarily secreted by M1 macrophages, promote the degradation of myofibrillar proteins and reduce protein synthesis, directly leading to muscle wasting. Consistent with the increase in M1 macrophages in old mice, the elevated serum levels of pro‐inflammatory cytokines were also decreased by treatment with GABA. In both the quadriceps and gastrocnemius muscles, the expression of COX‐2 and iNOS proteins was significantly higher in the OM control group than in the YM control group. However, GABA treatment reduced the expression of both inflammatory markers in old mice. These results indicate that treatment with GABA in old mice could improve the fundamental cause of sarcopenia by restoring muscle immune homeostasis through regulating macrophages and suppressing inflammatory responses.

**FIGURE 8:**
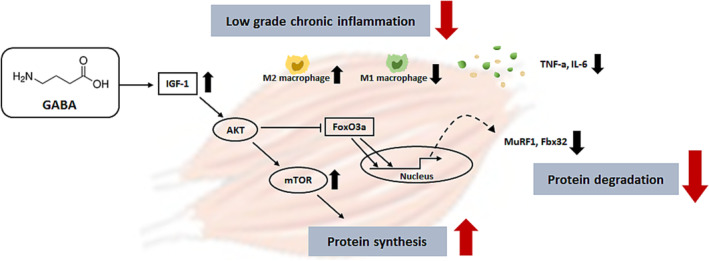
Schematic model for the mechanism of GABA in preventing sarcopenia through the regulation of protein degradation and inflammaging.

In conclusion, this study demonstrates that GABA improved sarcopenia by effectively inhibiting muscle protein degradation. It not only controlled muscle protein degradation in old mice but also regulated inflammaging by restoring the M1/M2 macrophage ratio (Figure 8). Through these effects, treatment with GABA on old mice aged 21 months or older for 7 weeks resulted in improvements in muscle strength, mass and fibre size. Therefore, we suggest that GABA can be used as a beneficial dietary supplement to prevent sarcopenia in the elderly.

## Conflicts of Interest

The authors declare no conflicts of interest.
